# ZnO@CdS Core-Shell Heterostructures: Fabrication, Enhanced Photocatalytic, and Photoelectrochemical Performance

**DOI:** 10.1186/s11671-016-1432-7

**Published:** 2016-04-18

**Authors:** Meng Ding, Nannan Yao, Chenggang Wang, Jinzhao Huang, Minghui Shao, Shouwei Zhang, Ping Li, Xiaolong Deng, Xijin Xu

**Affiliations:** School of Physics and Technology, University of Jinan, 336 Nanxinzhuang West Road, Jinan, 250022 Shandong Province People’s Republic of China

**Keywords:** ZnO@CdS, Heterostructure, Photocatalytic, Photoelectrochemical

## Abstract

ZnO nanorods and ZnO@CdS heterostructures have been fabricated on carbon fiber cloth substrates via hydrothermal and electrochemical deposition. Their photocatalytic properties were investigated by measuring the degradation of methylene blue under ultraviolet light irradiation. The result illustrated that the photodegradation efficiency of ZnO@CdS heterostructures was better than that of pure ZnO nanorods, in which the rate constants were about 0.04629 and 0.02617 min^−1^. Furthermore, the photocurrent of ZnO@CdS heterostructures achieved 10^2^ times enhancement than pure ZnO nanorods, indicating that more free carriers could be generated and transferred in ZnO@CdS heterostructures, which could be responsible for the increased photocatalytic performance.

## Background

Recently, semiconductor-based photocatalysts, as a kind of “green technology”, have attracted much more attention, in which varieties of metal oxide semiconductors, such as TiO_2_ [1–4], ZnO [5], SnO_2_ [6, 7], Cu_2_O [8, 9], CdS [10], and ZnS [11], have been fabricated as photocatalysts to decompose environmental pollutants. Among them, ZnO has been systemically investigated as photocatalysts due to its high electron mobility, flexible morphologies, easy synthesis, low cost, and nontoxicity [12, 13]. However, the inherent drawbacks of ZnO, including larger bandgap and fast internal recombination of photogenerated electron–hole pairs, result in low photodegradation efficiency [14, 15]. In addition, serious photocorrosion in the photocatalytic process also influences the degredation effect for organic pollutants. All the abovementioned have greatly hindered the potential applications. Many efforts have been put to improve the efficiency of photogenerated carriers and extend the spectral response range, such as doping [16], loading noble metals [17, 18], and combining with other semiconductors [19–30], and so on. Combining ZnO with other narrow bandgap semiconductors (for example ZnSe [19, 20], Cu_2_O [29], CdSe [24], CdS [21, 30] etc.) has proved to be feasible for promoting photocatalytic performance. Among these materials, CdS attracts much interest because of the similar lattice structures between CdS and ZnO. Moreover, ZnO/CdS heterojunction can induce a type-II band structure, the conduction band of ZnO is located between the valence band and the conduction band of CdS, which can hinder the recombination of photogenerated electron and hole. Xu et al. prepared the ZnO sheet-based hierarchical microspheres incorporated with CdS nanoparticles by hydrothermal method followed by ultrasonication treatment. The ZnO/CdS heterostructures exhibit higher photocatalytic activity than pure ZnO under sunlight [14]. Kundu reported a simple wet chemical route to obtain nanoscale heterostructures of ZnO/CdS without using any molecular linker, the heterostructures with the CdS loading exhibit high activity for the degradation of methylene blue (MB) under solar irradiation conditions, and also the photoactivity of the material could be tuned by manipulating the interface of the heterostructure [21]. Though some reports about ZnO@CdS heterostructure with enhanced photocatalytic activity have been reported, however, the efficiency of photocatalytic degradation needs further improvement. Furthermore, the photoelectrochemical performance of ZnO@CdS heterostructure grown on carbon fiber cloth with high photocurrent response is rarely reported.

Herein, ZnO@CdS heterostructures have been fabricated on carbon fiber cloth substrate by a two-step method including electrochemical deposition and hydrothermal method. The morphologies, structures, photocatalytic, and photoelectrochemical properties of as-grown ZnO and ZnO@CdS heterostructures were carefully investigated.

## Methods

### Materials Preparation

#### ZnO Nanorods

ZnO nanorods were grown on carbon fiber cloth by electrochemical deposition method. Firstly, carbon fiber cloth was cleaned by sonication in acetone, ethanol, and deionized water. Then, the mixed aqueous solution of 5 mM zinc nitrate hexahydrate (Zn(NO_3_)_2_ · 6H_2_O) and 5 mM hexamethylenetetramine (HMT) were used as the electrolyte, in which the carbon fiber cloth substrate, a 2 × 2 cm platinum plate, and Ag/AgCl in a saturated KCl solution were used as working electrode, counter electrode, and reference electrode, respectively. Finally, the electrolytic cell was placed in a water bath to keep constant temperature of 90 °C. The reaction was carried out for 1 h at a constant potential of −0.9 V versus the reference electrode. After reaction, samples were washed by deionized water several times, and dried in an oven at 60 °C for several hours.

#### ZnO@CdS Heterostructures

The CdS layer was synthesized on ZnO nanorods using hydrothermal method. Further, 114.2 mg cadmium chloride (CdCl_2_ · 2H_2_O), 114 mg thiourea (CH_4_N_2_S), and 49 mg polyethylene glycol (PEG) were dissolved into a given amount (40 mL) of deionized water. The mixture solution was transferred into a Teflon-lined stainless autoclave. Then, the as-grown ZnO nanorods on carbon fiber cloth substrate were put into autoclave. After that, the autoclave was sealed and maintained at 140 °C for 9 h, and cooled to room temperature naturally. The samples were taken out of the solution and washed with ethanol and deionized water several times, followed by drying at 60 °C in an oven for several hours.

### Material Characterizations

The morphologies and structures of as-grown ZnO nanorods and ZnO@CdS heterostructures were characterized by the field emission scanning electron microscopy (FESEM) (model: NoVa^TM^ Nano SEM 250, FEI Company), X-ray diffraction (XRD) (model: Bruker D8 Advance) and transmission electron microscopy (TEM) (model: Tecnai G2 F20, FEI Company). The surface chemical composition and states of the ultimate ZnO@CdS heterostructure was analyzed using an X-ray photoelectron spectrometer equipped with a monochromatic Al Ka source (1486.6 eV) (model: Thermo ESCALAB 250XI).

### Photocatalytic Activity

Photocatalytic activities were tested by the photodegradation of methylene blue (MB) with photocatalytic reaction apparatus (XPA series-7, Nanjing) equipped with a 500-W mercury lamp as the light source. Typically, the sample (ZnO nanorods and ZnO@CdS nanomaterials) grown on carbon fiber cloth substrate (2 × 1.5 cm) as photocatalyst was placed into a quartz tube filled with 5 mL of MB (5 mg/L) aqueous solution. The solution was kept for 60 min in the dark to ensure the adsorption–desorption equilibrium between photocatalyst and methylene blue, and then irradiated with UV irradiation. The photocatalytic degradation of MB dye was analyzed by measuring the absorbance at 664 nm in the presence of photocatalyst exposed at different irradiation time intervals with a UV-Vis spectrophotometer (TU-1900/1901, Beijing). The photocatalytic measurements of the three photocatalysts (ZnO and ZnO@CdS) were performed in three independent experiments. All the experiments were performed at room temperature.

### Photoelectrochemical Characterization

All electrochemical measurements were performed using a typical three-electrode system, which the sample grown on carbon fiber cloth substrate, a 2 × 2 cm platinum plate, and Ag/AgCl in a saturated KCl solution were used as working electrode, counter electrode, and reference electrode, respectively. Further, 0.5 M Na_2_SO_4_ aqueous solution (with pH buffered to ∼7.0) was used as the electrolyte. A 300-W Xe lamp was used as the light source for photocurrent test.

## Results and Discussion

**Fig. 1:**
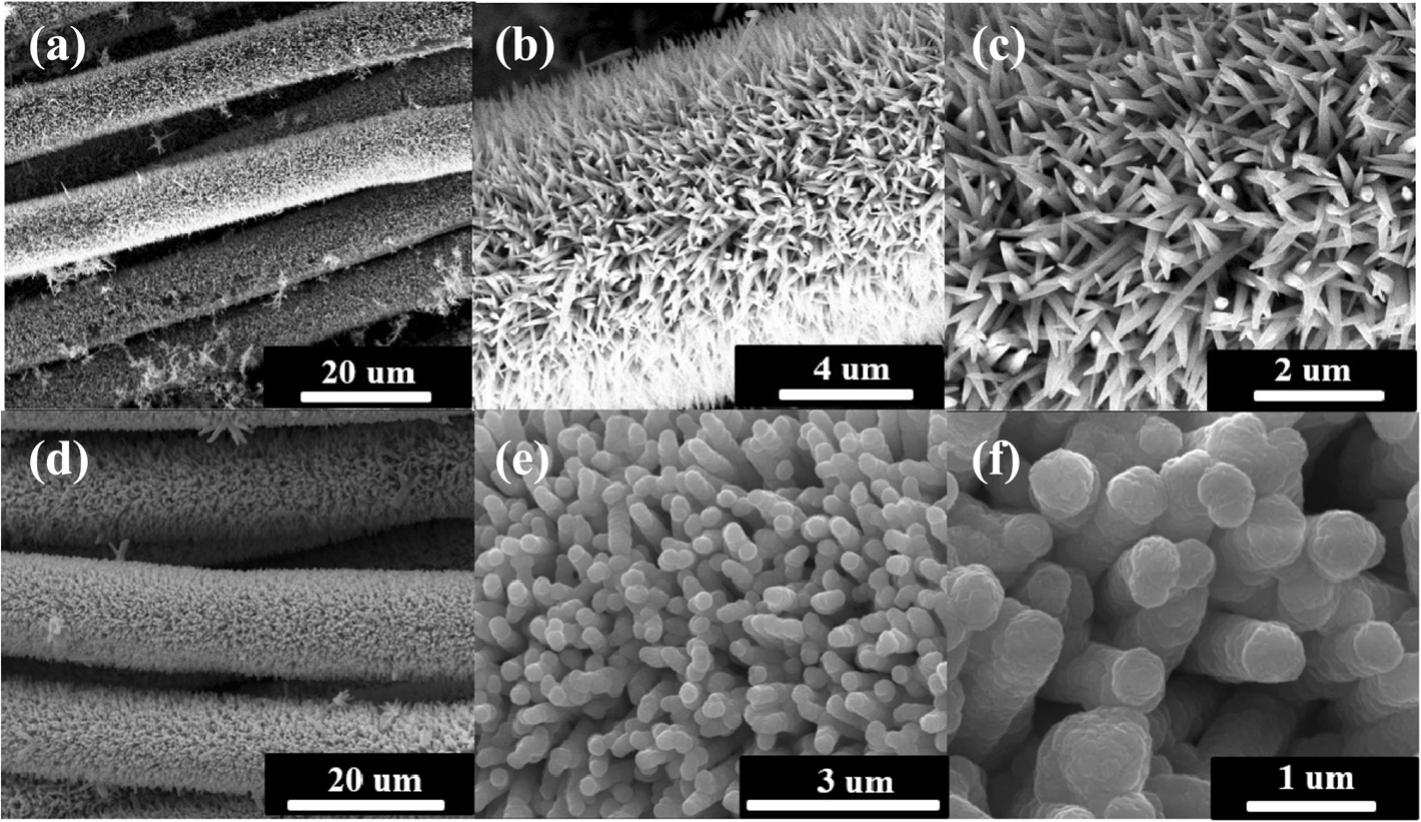
FESEM images of pure ZnO nanorods (**a**–**c**) and ZnO nanorods/CdS composites (**d**–**f**) grown on carbon fiber cloth at different magnifications

Typical FESEM images of pure ZnO nanorods and ZnO@CdS nanocomposites grown on carbon fiber cloth with different magnifications are shown in Fig. 1. ZnO nanorods are observed synthesized in a large yield on substrate with uniform density and size (Fig. 1a, b). The high-magnified SEM image (Fig. 1c) clearly shows that the diameter of the ZnO nanorod is about 100 nm. SEM images observed from ZnO@CdS heterostructures are shown in Fig. 1d–f. Compared with pure ZnO nanorods, it can be seen that the average diameter of nanorods becomes thicker and the tips are not as sharp as the nanorods; furthermore, the surfaces become rough.

**Fig. 2:**
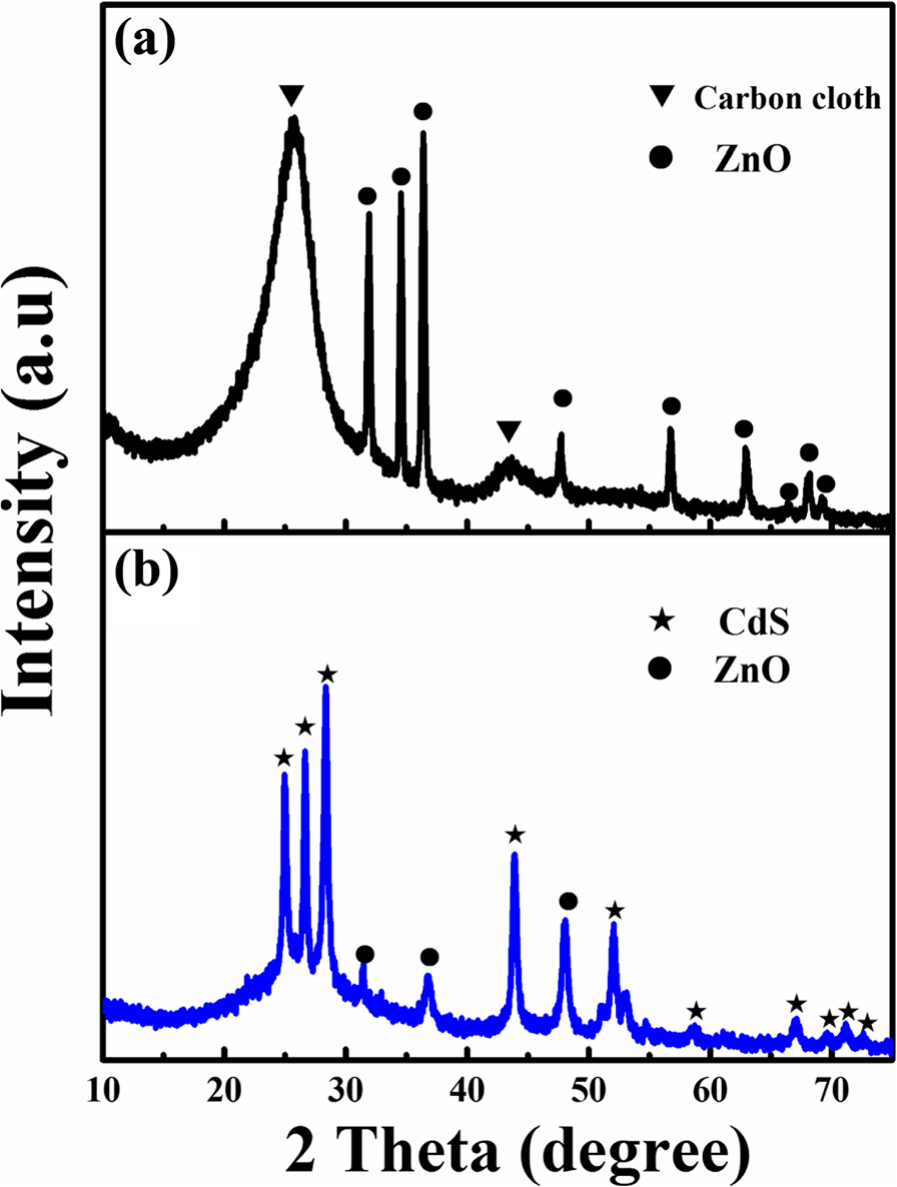
XRD patterns of ZnO and ZnO@CdS heterostructure. Peaks corresponding to ZnO and CdS are labeled

The typical XRD spectrum of the as-grown ZnO nanorods and ZnO@CdS nanocomposites grown on carbon fiber cloth are depicted in Fig. 2. Figure 2a shows the XRD pattern of as-grown ZnO nanorods, in which the broad peaks located at 25.7° and 43.7° are ascribed to the diffraction peak of the carbon cloth. All the diffraction peaks at 31.8°, 34.4°, 36.3°, 47.7°, 56.7°, 63.0°, 66.4°, 68.1°, and 69.3° can well be attributed to the crystal planes (100), (002), (101), (102), (110), (103), (200), (112), and (201) of ZnO, which indicates that the products can be indexed to hexagonal wurtzite structure of ZnO without any impurities (JCPDS:79-0205). ZnO@CdS heterostructures exhibited new diffraction peaks centered at 24.9°, 26.6°, 28.3°, 43.9°, 52.1°, 58.6°, 67.1°, 69.6°, 71.2°, and 72.7°, corresponding to the crystal planes (100), (002), (101), (110), (112), (202), (203), (210), (211), and (114) of the hexagonal phase of CdS (JCPDS:80-0006) (shown in Fig. 2b). The result illustrated that ZnO@CdS nanocomposites were composed of a hexagonal structure ZnO and CdS. Moreover, no crystal phase transformation of ZnO was observed after CdS coating, confirming that the obtained product was of high purity.

**Fig. 3:**
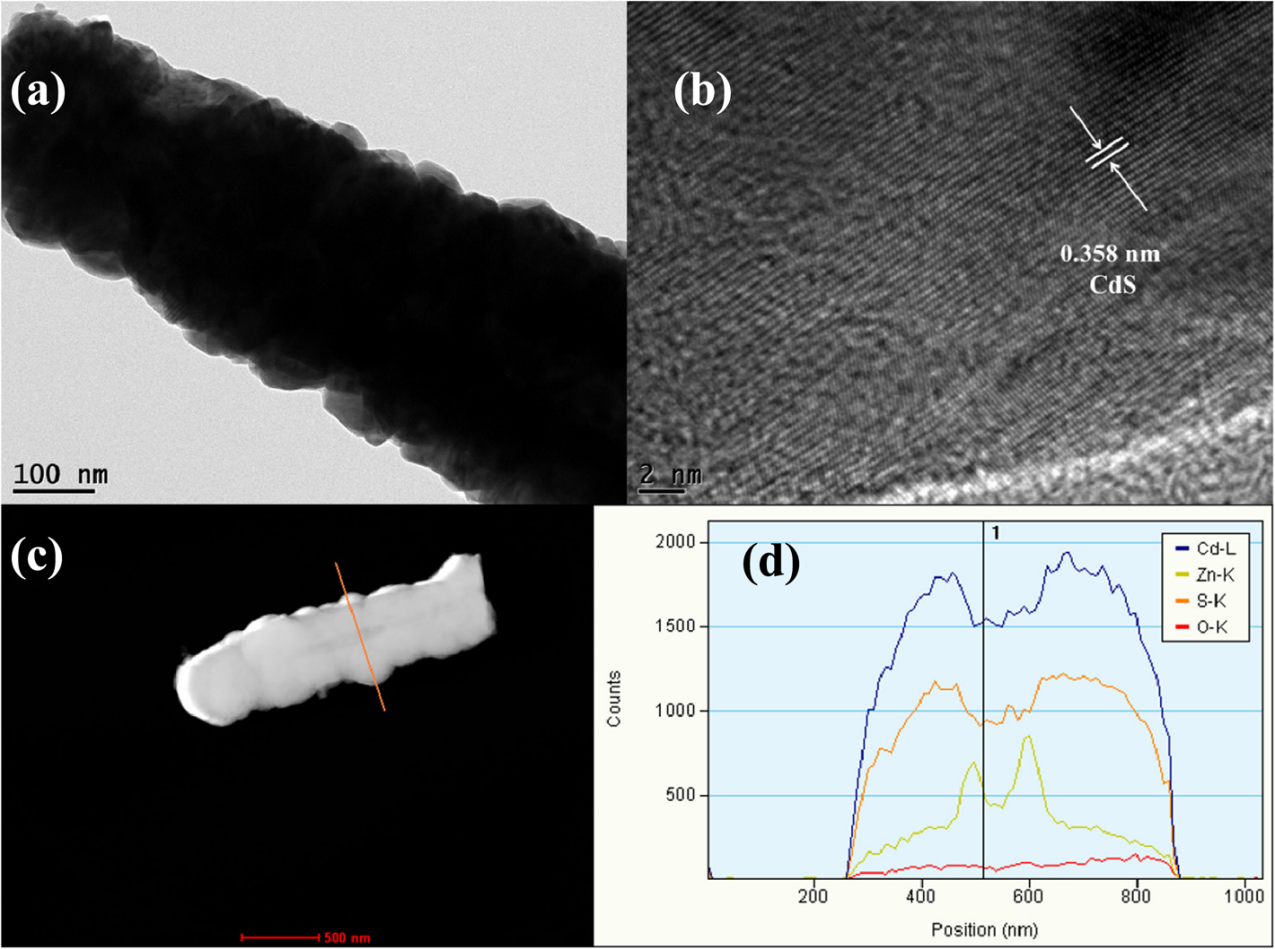
**a** TEM image and **b** a high-resolution TEM image of ZnO@CdS heterostructure. **c** Analyzed area of EDX line scanning analysis. **d** EDX line scanning profiles across ZnO@CdS heterostructure indicated in (**c**)

TEM and HRTEM measurements were carried out to further investigate the structure of ZnO@CdS heterostructure. The rod-like morphologies with the diameters of about 200–300 nm were observed from ZnO@CdS sample (Fig. 3a). The HRTEM image in Fig. 3b displayed that the well-resolved two-dimensional lattice fringes are about 0.358 nm corresponding to the interplanar space of (100) plane of hexagonal wurtzite CdS [31], which indicated that the outer shell was CdS. Figure 3d shows line scan spectra acquired across the single nanorod. The intensities of the curves further proved that the core-shell structure was formed with multiple shells.

**Fig. 4:**
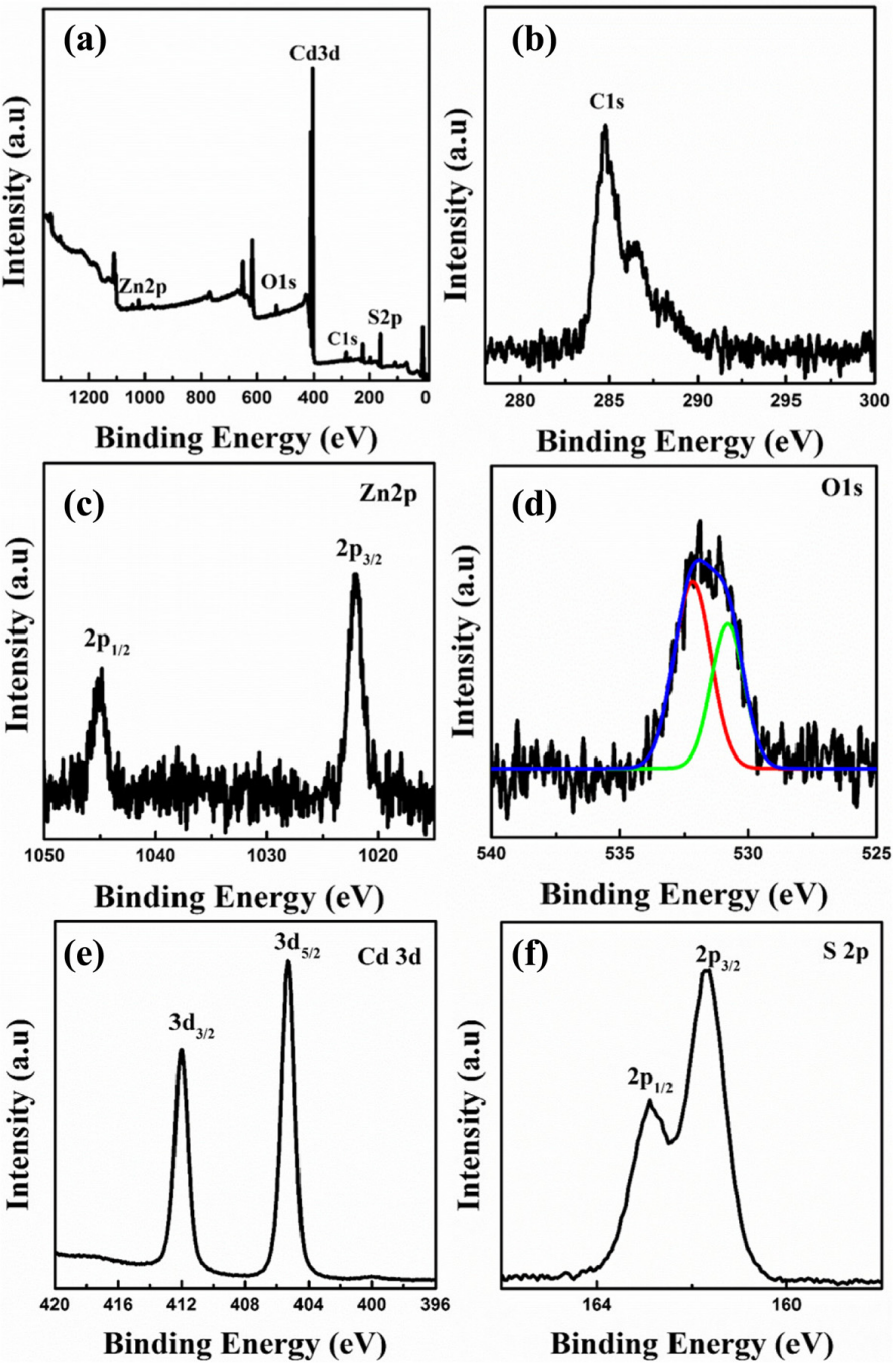
XPS spectra of ZnO@CdS heterostructure. **a** Survey spectra, **b** C1s, **c** Zn 2p, **d** O 1 s, **e** Cd 3d, and **f** S 2p, respectively

X-ray photoelectron spectroscopy (XPS) spectra were recorded (Fig. 4) to further study the surface composition and chemical states of ZnO@CdS heterostructure. Figure 4a shows the typical XPS survey spectra, four kinds of elements, Zn, O, Cd, S were observed, which is in agreement with the XRD results. The C1s peak was taken as a standard reference with a binding energy of 284.6 eV (as shown in Fig. 4b) and mainly came from the hydrocarbon contaminants, which normally resided in XPS spectra [14]. The XPS peaks (Fig. 4c) at binding energies of about 1022.1 and 1044.8 eV were assigned to the Zn 2p_3/2_ and Zn 2p_1/2_ states [24], which suggested that Zn element presented in the form of Zn^2+^ in products. The asymmetric O 1s state for ZnO@CdS heterostructure in Fig. 4d could be fitted into two peaks. The peak located at 530.8 eV was ascribed to Zn−O bonds of ZnO [32], while the energy peak at 532.2 eV was attributed to the adsorbed O_2_ or surface hydroxyl species [33]. The position of Cd 3d_5/2_ and Cd 3d_3/2_ peaks were at about 405.3 and 412.0 eV (Fig. 4e), which is in agreement with the previous report for CdS [24]. The peaks depicted in Fig. 4f at about 161.6 eV and 162.7 eV could be attributed to S 2p_3/2_ and S 2p_1/2_, which were ascribed to the hybrid chemical bond species of S^2−^ and Cd−S [24, 34].

**Fig. 5:**
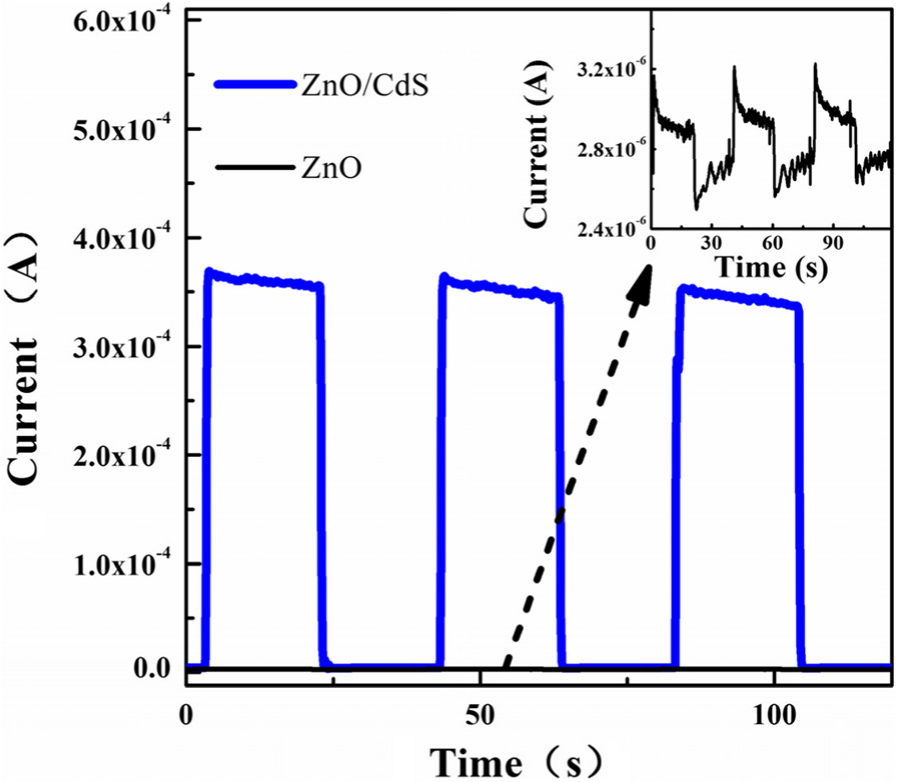
Photocurrent response of ZnO and ZnO@CdS heterostructure under Xe lamp irradiation. In the *inset* is the photocurrent response of ZnO nanorods

The photoelectrochemical performance of ZnO nanorods and ZnO@CdS heterostructure were conducted to study the separation efficiency of charge carriers. The corresponding photocurrent responses to several light on-off cycles are shown in Fig. 5. The fast and uniformly photocurrent responses implied that the charge transport in samples was very quick. In the dark, the current response for ZnO@CdS heterostructure could be negligible due to the small value (about 10^−6^ A). In the case of light illumination, the photocurrent increased sharply, as high as 10^−4^ A. It also decreased quickly as soon as the light illumination turned off. In addition, the photocurrent of ZnO@CdS heterostructure achieved 10^2^ times enhancement compared to that of pure ZnO nanorods (about 10^−6^ A), as shown in the inset of Fig. 5. Kuang et al. reported that with ZnO@CdS/CdSe porous nanotube arrays with a unique porous nanotube structure and cosensitization effect, photoelectrochemical water-splitting performance was improved than that of pure ZnO. The single-shelled ZnO@CdS acquires an increase of 36.4 time compared to the value for ZnO. In our experiment, the increasing ratio (about 10^2^ times) is much larger compared with previous reports [24]. It was deduced that more free carriers could be generated and transferred in ZnO@CdS heterostructure leading to high separation efficiency than that of ZnO nanorods, resulting in the increasing photocurrent.

**Fig. 6:**
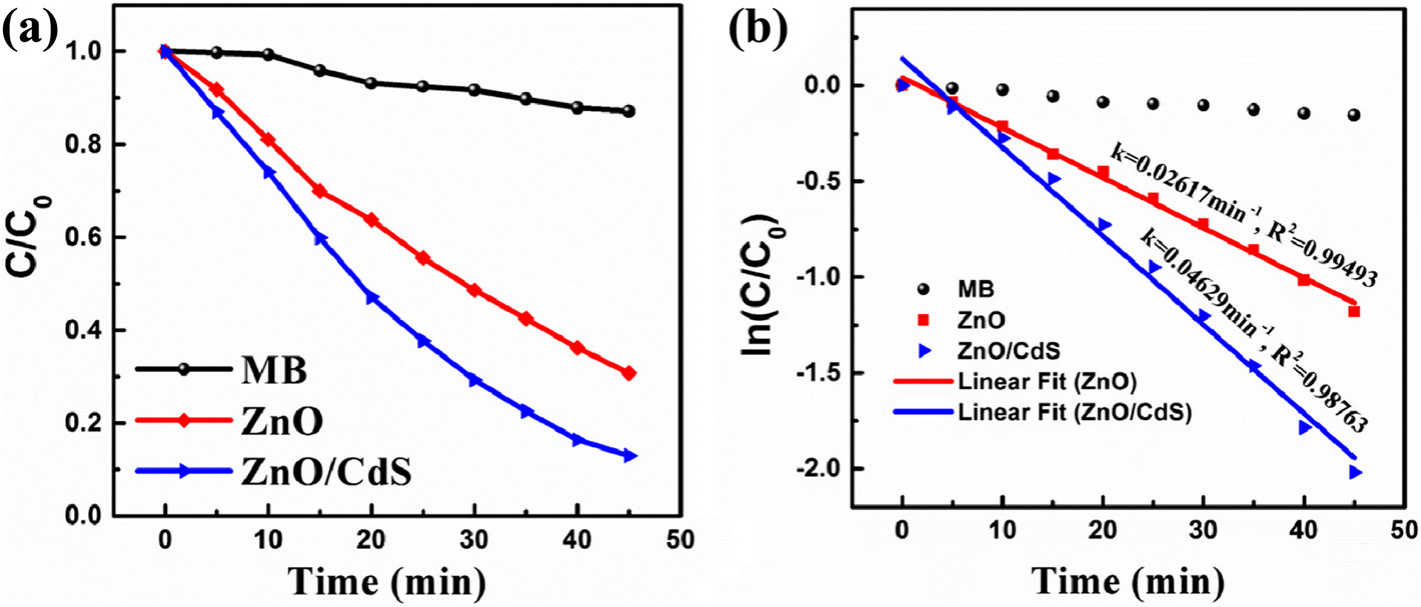
**a** Relative concentration (C/C_0_) of MB versus time under visible light irradiation using as-grown ZnO and ZnO@CdS heterostructure as photocatalysts. **b** The corresponding plots of –ln(*C*_t_/*C*_0_) versus irradiation time

**Fig. 7:**
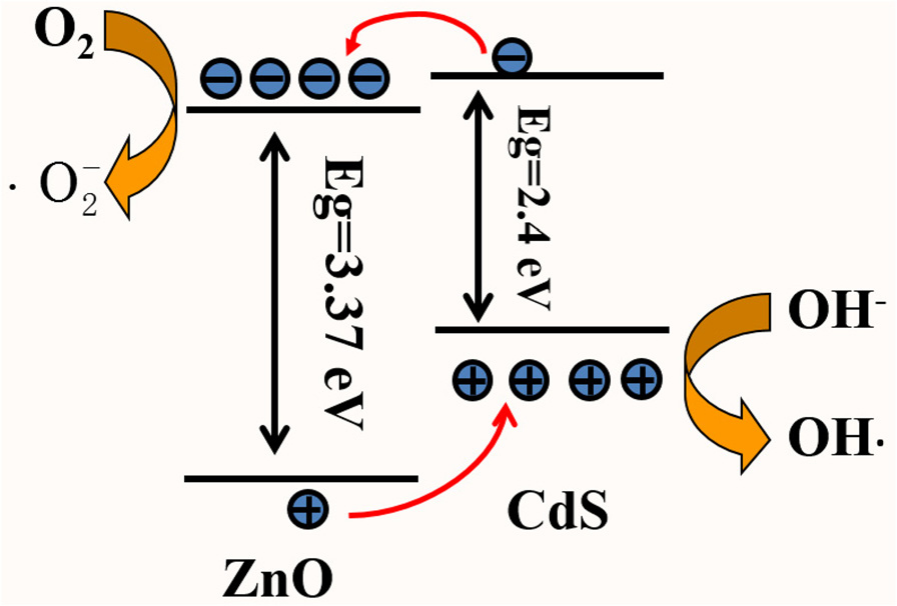
The schematic profile exhibiting the energy band alignment between ZnO and CdS

Figure 6a depicts the photocatalytic activities of as-prepared samples for the degradation of MB aqueous solution. It can be seen that the photodegradation efficiency of MB has almost no change in the absence of photocatalysts. However, once the photocatalysts were added into MB aqueous solution, the photodegradation efficiencies were significantly enhanced. Meanwhile, the photodegradation activity of ZnO@CdS heterojunction was much higher than that of pure ZnO. The photodegradation rate constant of MB versus degradation time was used to compare the photodegradation property intuitively, which were estimated by the pseudo-first-order kinetics model as the following [9]:

$$ \ln \left(\frac{C}{C_0}\right)=-kt, $$

where *C*_0_ represents the initial concentration of MB and *C* refers to the concentrations at different irradiation time *t*, and *k* is the reaction rate constant. The linear transform of ln(*C*/*C*_0_) versus time *t* of MB photodegradation over ZnO and ZnO@CdS is shown in Fig. 6b. The rate constant (*k*) was evaluated by the slopes of linear fit for each photocatalytic reaction. The observed rate constant was about 0.04629 min^−1^ for ZnO@CdS heterojunction, which was obviously higher than 0.02617 min^−1^ for pure ZnO nanorods. Compared with the previous reports, the photodegradation rate is obviously improved. The experimental results indicated that the addition of CdS layer on ZnO nanorods could facilitate charge transfer thus significantly improving the photocatalytic activities. The mechanism for highly efficient carrier separation and transport at the interface of the ZnO@CdS heterostructure was proposed according to the result of photoelectrochemical test and photodegradation experimentation, which is similar with the previous reports [21, 26, 29, 30, 35]. Figure 7 displays the type-II band alignment of ZnO@CdS heterostructure and the mechanism of the photocatalytic reaction, which included the electron–hole pair generation by incident photons, separation and transport of photogenerated carriers, and reduction/oxidation reactions of the absorbed species. This band alignment was beneficial to fast separation and transport of photogenerated holes and electrons at the interface of the of ZnO@CdS heterostructure. Therefore, ZnO@CdS heterostructure exhibited superior photocatalytic performance purer than that of ZnO.

## Conclusions

In summary, ZnO@CdS heterostructure was successfully fabricated on carbon fiber cloth substrate by a simple two-step method including electrochemical deposition and hydrothermal method. The photoelectrochemical measurement proved that more free carriers could be generated and transferred in ZnO@CdS heterostructure leading to high separation efficiency than that of ZnO nanorods. The type-II band structure of ZnO@CdS could improve the efficiency of carrier separation and transport; thus, the photocatalytic activities and photoelectrochemical could be significantly enhanced by the introduction of CdS layer on ZnO nanorods.
